# Clinical efficacy of electroacupuncture-like magnetic therapy compared to conventional transcutaneous electrical nerve stimulation in individuals with carpal tunnel syndrome

**DOI:** 10.1038/s41598-023-46916-0

**Published:** 2023-11-16

**Authors:** Sui-Foon Lo, Li-Wei Chou, Huynh Chung, Hsiu-Chen Lin

**Affiliations:** 1 School of Chinese Medicine, College of Chinese Medicine, China Medical University, Taichung, Taiwan, ROC; 2 Department of Physical Medicine and Rehabilitation, China Medical University Hospital, Taichung, Taiwan, ROC; 3 Department of Physical Therapy, Institute of Rehabilitation Science, China Medical University, No. 100, Sec. 1, Jingmao Rd., Beitun Dist., Taichung City, 406040 Taiwan, ROC; 4 Department of Physical Medicine and Rehabilitation, Asia University Hospital, Asia University, Taichung, Taiwan, ROC

**Keywords:** Health care, Rheumatology

## Abstract

This study aimed to evaluate the clinical efficacy of an electroacupuncture-like magnetic therapy (ELMT) and conventional transcutaneous electrical nerve stimulation (TENS) in patients with carpal tunnel syndrome (CTS). A prospective randomized controlled trial in single-centre was conducted. Thirty-four CTS patients confirmed by electrodiagnostic study were randomized into TENS or ELMT group and completed a six-week treatment program. TENS or ELMT treatment was applied on acupuncture point PC-6 (Neiguan) and one selected hand acupoint. Therapeutic exercises were also included after the electrophysical modality. Their physical signs, motor and sensory performances, Boston Carpal Tunnel Questionnaire (BCTQ) scores, and results of electrodiagnostic study were evaluated. After treatments, both groups demonstrated significantly decreased BCTQ scores and positive rate of Tinel’s sign in the major symptomatic side, which indicated improvements in the symptom severity and physical functions. Significant increases in distal sensory amplitude and nerve conduction velocity of the median nerve were only found in the ELMT group. Our study found either conventional TENS or ELMT plus therapeutic exercises could improve the symptomatology and physical provocation sign of CTS. The ELMT has additional improvement in the nerve conduction in patients with CTS.

Carpal tunnel syndrome (CTS) is a common entrapment neuropathy, and arises from compression of median nerve at the wrist^1^. In survey among US adults, CTS occurs most often between the age of 35 to 54 and the overall prevalence was higher in females than males and higher in White than non-White. In general population classified as ever worked, the prevalence of self-reported CTS was 1.55%^2^. In a population-based cohort study using data of National Health Insurance Research Database in Taiwan, the annual incidence of CTS was approximately 0.4% during a decade from 2003 to 2012^3^. Middle age females seemed still to have the highest incident rate.

CTS diagnosis is often based on clinical symptoms, physical signs, electrophysiological measurements, or image study^2,4^. With the progression of the disease, hand muscles weakness and atrophy may occur^1^. CTS affects patients significantly on hand grip and opposition functions as well as quality of life^5,6^. The treatment of CTS has undergone progressive change over the years to alleviate symptoms. Conservative treatment for CTS includes various strategy alone or in combination. Therapies like steroid, non-steroidal anti-inflammatory drugs, platelet-rich plasma injections, vitamin B6; splint use, activity alteration, Kinesio taping, tendon/nerve gliding exercise; and electrotherapy, therapeutic ultrasound, extracorporeal shockwave therapy and acupressure; have been suggested over the years^7–9^. Transcutaneous electrical nerve stimulation (TENS) is one of the typical electrophysical modalities used in clinics, and is beneficial for various pain syndromes^10–12^.

Many patients with neuropathic pain would seek for the assistance of complementary and alternative medicine (CAM) therapies, such as megavitamins, magnet therapy, acupuncture, herbal remedies, and chiropractic manipulation^13^. Magnetic therapy is also a type of electrophysical modalities, which uses magnetism or magnetic field for therapeutic purposes, safe and easy to apply with the effects of analgesia, anti-inflammation and tissue healing. The postulated mechanism of magnetic therapy is at the cellular level. Magnetism activates the metabolic transfers, enzymatic processes and cell membrane function, and thus positively affects the immune system and autonomic nervous system, causing dilation of blood vessels and removing pain-inducing toxins^14,15^. It is commonly assumed that analgesic effects can be induced by hormone with analgesic property, such as endorphin or by changing membrane’s permeability of neuron. Magnetic therapy is recommended for treatment of muscular-skeleton system, vascular, immune, and endocrine disorders^16^. Use of time-varying pulsed electro-magnetic fields (PEMF) to treat patients with CTS showed effectiveness in pain reduction and improvement in objective neuronal functions^17,18^.

Acupuncture is another common choice of the CAM therapies^19^. Our previous study showed the effectiveness of acupuncture on improved symptom, grip strength, electrophysiological function for CTS patients; while combined with 0.8 mA, 2 Hz current, electroacupuncture also showed improvement in symptom^20^. The systematic review also confirmed the positive effects of acupuncture or electroacupuncture in symptom relief and function improvement^21^. However, the needle puncture and safety concern of acupuncture may limit the patients’ acceptance of the treatment, and thus facilitate the development of modern acupuncture-like stimulation methods since 1950s^22^. Among the modern acupuncture-like stimulation methods, only the uses of low-level laser therapy and microamperes TENS to stimulate acupuncture points have been studied on eleven CTS patients and showed effective in pain relief^23^. The aim of this study is to investigate the effectiveness of an electroacupuncture-like magnetic therapy (ELMT) and compared with conventional TENS in patients with symptomatic CTS.

## Methods

### Participants and design

The design of this study is as a prospective randomized, single-center, interventional clinical trial with two parallel assignment groups. This study has been registered on ClinicalTrials.gov, and the Identifier is NCT01277003 (14/01/2011). The potential participants were patients who visited our outpatient clinics with symptoms that suggested CTS.

#### Inclusion criteria

In this study, CTS was diagnosed clinically by senior physicians with at least one of the symptoms of numbness, tingling, or pain in the wrist or hand. The diagnosis was further confirmed with electrodiagnostic studies, which indicated at least one of the following criteria^20^: (1) prolonged distal motor latency (≧ 4.5 ms) to the abductor pollicis brevis muscle, stimulation at the wrist, 8 cmproximal to the active electrode; (2) prolonged orthodromic index-wrist peak sensory latency (≧ 3.5 ms) stimulation at the index at 14 cmdistal to the active electrode at the wrist, and decreased orthodromic palm–wrist sensory nerve conduction velocity (P–W SNCV < 35 m/s) stimulation in the palm at a distance of 8 cm distal to the wrist; or (3) decreased orthodromic index–wrist sensory nerve conduction velocity (< 40 m/s) at a distance of 14 cm and P–W SNCV < 35 m/s.

#### Exclusion criteria

The medical and surgical conditions that predispose one to peripheral neuropathy such as hypothyroidism, gout, systemic lupus erythematosus, rheumatoid arthritis, diabetes mellitus, chronic renal failure; the presence of disorders such as cervical spondylosis, polyneuropathy, that might cause numbness in the hand; treatment with steroids or nonsteroidal anti-inflammatory drugs within the previous month; and steroid injection within the previous 6 months; any surgery for peripheral nerve in the upper limb such as carpal tunnel surgical release, history of trauma in the upper limb such as wrist fracture, or having pregnancy.

During the one-year recruiting period, patients with electro-diagnostically confirmed CTS were invited to participate in our study. Possible beneficial and side effects were fully explained before allocation to the treatments. Informed consent, as approved by the local ethics committee, was obtained from every enrolled participant before the study. This research was performed in accordance with the standards of ethics outlined in the Declaration of Helsinki.

### Baseline testing and outcome measurement

The participant would be first evaluated using the BCTQ^5^ to assist CTS evaluation of the Symptom Severity (SS) and the Functional Status (FS) scales, administered with the validated the Hong-Kong Chinese version^24^. Their age, sex, body weight, height, education, and pertinent medical history, physical examinations (Tinel’s sign and Phalen test) and motor and sensory performances (grip and pinch strength, and two-point discrimination) were administered and recorded. The side with more severe symptoms and less severe symptoms was defined as the major symptomatic side and the minor symptomatic side respectively. Electrodiagnostic studies were performed the same as our previous study^20^, adhering to the uniform operating protocol of the electrodiagnostic machine (NEuropack MEM3202) in a laboratory, with a controlled ambient room temperature of 25 °C. Sensory and motor nerve conduction studies of the median and ulnar nerves were conducted using surface electrodes for stimulating and recording. Latencies were measured from the time of stimulus to the onset or peak responses for motor or sensory conduction studies, respectively.

### Allocation

**Figure 1:**
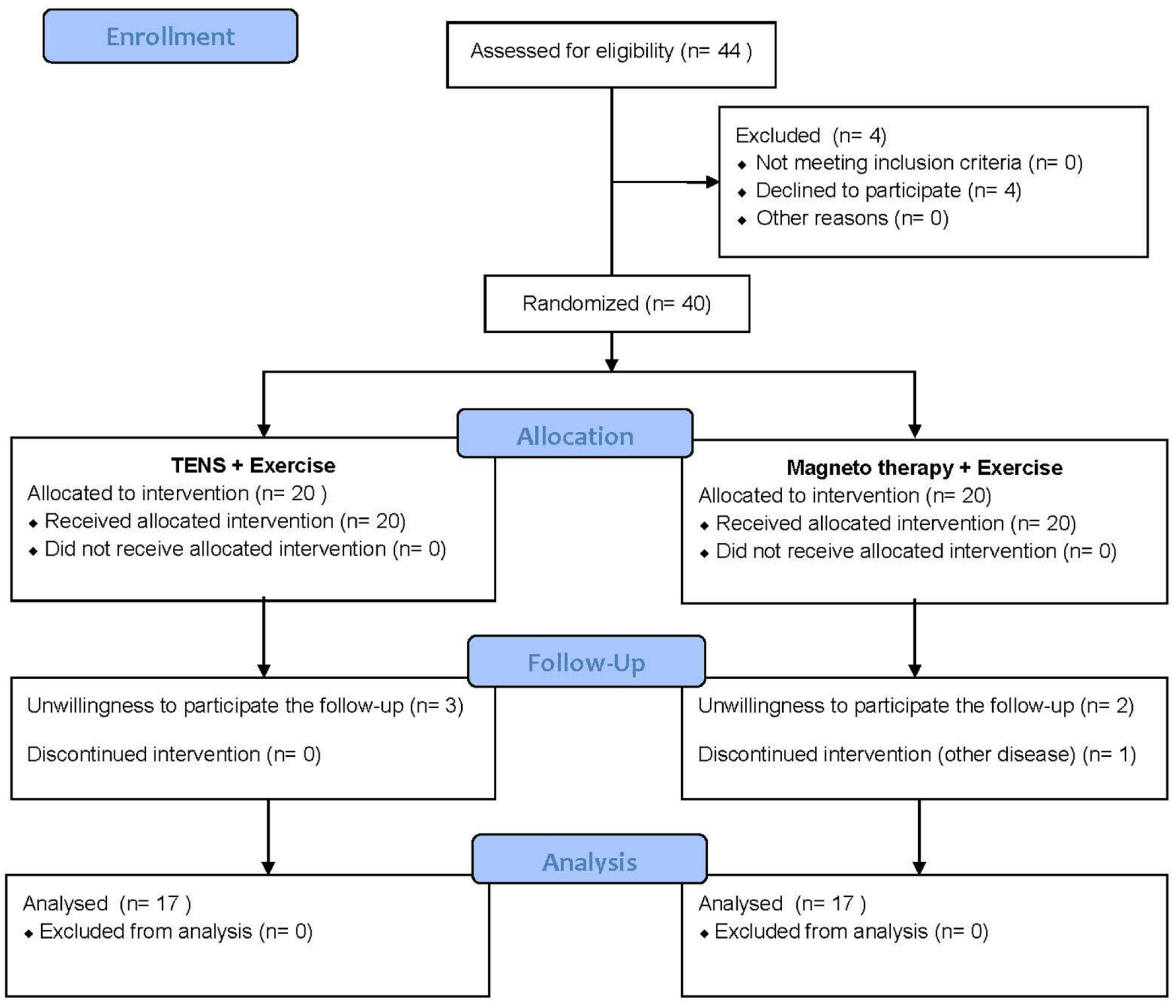
The flow chart of the study.

We then randomly assigned the participants into two treatment groups, but tried to evenly distribute them to be matched with similar demographic characteristics and CTS severity. One group received conventional TENS (TENS group) and another group received ELMT with Aculife (ELMT group). Forty patients with carpal tunnel syndromes were screened and enrolled into this study, they were evenly allocated into either TENS or ELMT group (Fig. 1).

### Interventions

All the assessment and the treatment were carried out in the outpatient clinics of the Department of Physical Medicine and Rehabilitation of our hospital. The intervention program would last for six weeks, one treatment session each day, 5 sessions per week. Each session consisted of one electrophysical modality treatment and exercises. Apart from different modalities, participants in both groups would all be instructed to perform tendon gliding and nerve gliding exercises for 15 min^25^ after either electrophysical modality treatment. The treatments could be performed on one or both hands of the patient, and the outcome measure would focus on the major symptomatic side.

#### Electroacupuncture-like magnetic therapy treatment

**Figure 2:**
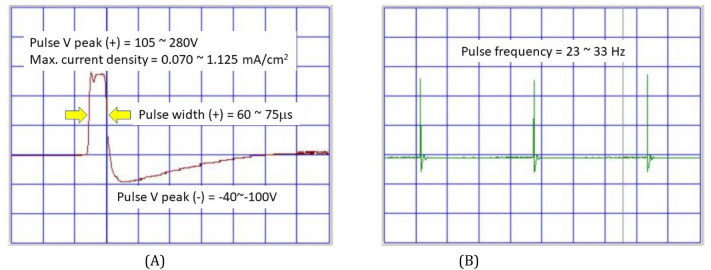
The characteristics of the pulsed electro-magnetic field (PEMF) delivered by the device (Aculife Magnetic Wave device, SMW-A01, Innohealth Technology Co., Taiwan) used in the magnetic therapy group. (**A**) single pulse; (**B**) multiple pulse. The actual output values (showing the range in figure) may be different depending upon the output adjustments (intensity, power mode) as well as the individual skin impedance.

**Figure 3:**
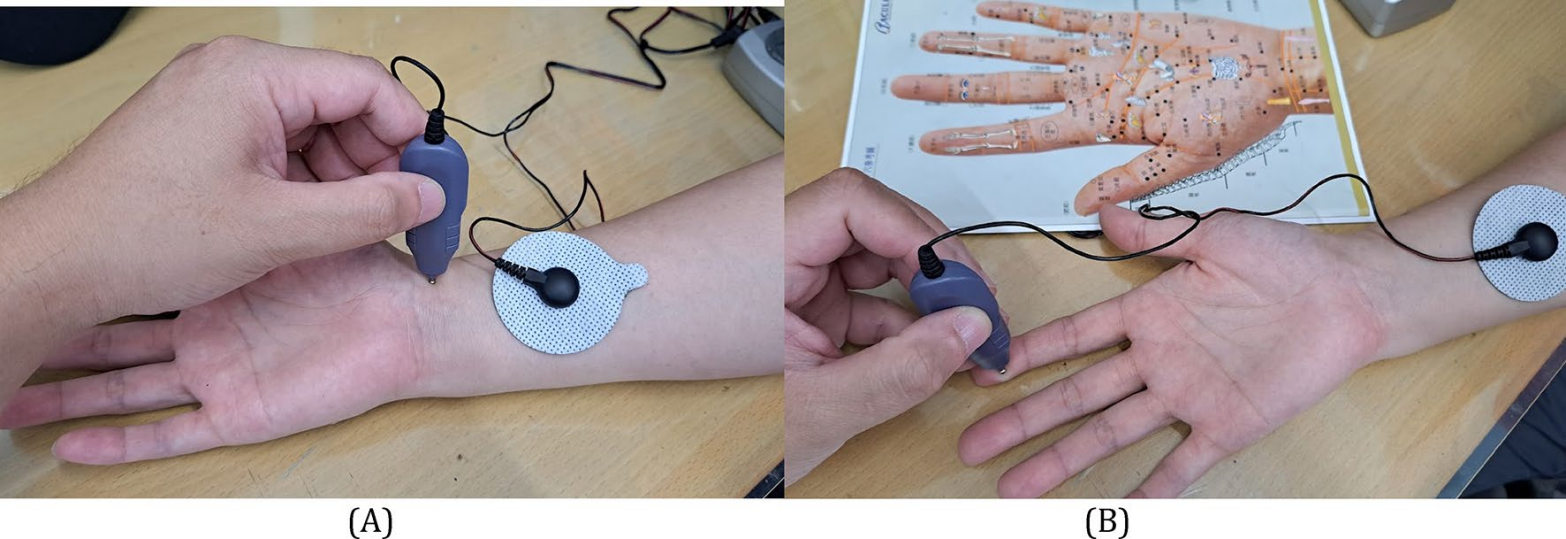
The acupuncture points selected to treat (**A**) acupoint PC-7 (Daling, in pericardium meridian); (**B**) hand acupuncture point of the right wrist.

The ELMT treatment was delivered with a magnetic therapy device (Aculife Magnetic Wave device, SMW-A01, Innohealth Technology Co., Taiwan). It is designed to deliver surface acupuncture-like stimulation, using a biphasic pulse with one high-voltage square phase in positive direction and then quickly changed in polarity and form a balanced negative triangular ramp, create time-varying PEMF (Fig. 2). The lead setup was one self-adhesive round electrode (5 cm radius) on acupoint PC-6 (Neiguan, in pericardium meridian) and the other metal pointer electrode would on two selected acupoints, the acupoint PC-7 (Daling, in pericardium meridian) and the hand acupoint at lateral side of the distal pulp of index, the hand acupuncture point of the wrist (Fig. 3). Each of the two acupoints would receive 15-min PEMF respectively, and every patient would receive 30- or 60-min treatment for one or both upper limbs daily.

#### Conventional TENS treatment

A typical TENS machine was used (GEM-STIM GM3A5XT, Chi-Mao Co., Taiwan) to delivered conventional TENS treatment. Two adhesive round electrodes (2.5 cm radius) were applied to the same treatment positions, as described above. Negative electrodes were placed on the carpal ligament (covering PC-6), and positive electrodes were placed on the distal pulp of index (covering the hand acupuncture point of the wrist). The device delivered asymmetric biphasic pulse current, and the parameter was set at a pulse rate of 30 Hz frequency and a stimulation period of 70 μs, which were similar to the AcuLife device. The device provides two channels output and enable bilateral treatment simultaneously. One TENS session lasted for 15 min, and at least a total of 30 sessions (5 sessions per week) were completed.

### Statistical analysis

Boston Carpal Tunnel Questionnaire (BCTQ) scores, motor and sensory performances, provocation signs, and electro-diagnostic measurements were evaluated before and after the treatment program to measure the treatment effectiveness. Statistical Package for the Social Sciences software (IBM Corp., Armonk, NY, USA) was used for statistical analyses. Independent T-test and Chi-square were used to compare continuous variables and category variables, respectively, of demographic characteristics and outcome variables of two groups. Paired T-test was used to examine the pre- and post-treatment effects in each group. Mann–Whitney U test was used for nonparametric variables. Statistical significance was considered when *p*-value < 0.05.

### Ethical approval

This research was reviewed and approved by the institutional review board of China Medical University Hospital (Protocol No. DMR99-IRB-223), and has been registered on ClinicalTrials.gov (Identifier: NCT01277003, 14/01/2011). This study was performed in accordance with the standards of ethics outlined in the Declaration of Helsinki. Informed consent was obtained from all participants.

## Results

At the end of the study, 17 participants (13 females, 4 males) in the TENS group and 17 (15 females, 2 males) in the ELMT group completed the treatment programs (Fig. 1).

### Demographic characteristics

There were no significant differences between groups in the baseline characteristics of demography including age, gender, body mass index (BMI), duration of disease, side of hand and the severity (*p* > *0.05*, Table 1).

**Table 1:** Demographic characteristics of two groups.

	TENS group Mean ± SD (%)	Magnetic therapy group Mean ± SD (%)
Number of patients	17	17
Gender (Female/Male)	13/4	15/2
BMI (kg/m^2^)	25.03 ± 3.78	25.62 ± 3.44
Age (years)	46.3 ± 11.0	47.4 ± 7.1
Duration of disease, frequencies (%)		
< 6 months	3 (17.6)	5 (29.4)
> 6 months	14 (82.4)	12 (70.6)
Side of hand, frequencies (%)		
Right	12 (70.6)	13 (76.5)
Left	5 (29.4)	4 (23.5)
Severity, frequencies (%)		
Mild	2 (11.8)	4 (23.5)
Moderate	9 (52.9)	7 (41.2)
Severe	1 (5.9)	3 (17.6)
Very severe	5 (29.4)	3 (17.6)

*BMI* body mass index; *SD* standard deviation; all *p*-value > 0.05 were not showed in the table.

### Questionnaire scores and physical signs

There was no significant difference between two groups with respect to improvement in BCTQ scales (*p* > 0.05). Significant differences were found compared before and after the intervention program for the two questionnaire scores in both groups (Table 2). Mean differences ± SD were 3.29 ± 6.37 in BCTQ-SS scale (*p* = *0.049*), 3.18 ± 5.90 in BCTQ-FS scale *(p* = *0.041)* for TENS group; and were 5.41 ± 7.60 in BCTQ-SS scale *(p* = *0.010)*, 2.71 ± 5.16 in BCTQ-FS scale *(p* = *0.046)* for ELMT group. This difference was also observed in the result of Tinel’s sign in TENS group *(p* = *0.004)* and ELMT group *(p* = *0.003)*.

**Table 2:** Boston Carpal Tunnel Questionnaire scores, motor and sensory performances and physical signs outcomes at baseline and follow up at 6 weeks.

	TENS group Mean ± SD		*p*-value within TENS group	ELMT group Mean ± SD		*p*-value within magnetic therapy group
	Baseline	Follow up		Baseline	Follow up	
BCTQ—Symptom	22.24 ± 8.21	18.94 ± 6.45	0.049*	24.18 ± 8.44	18.76 ± 7.30	0.010*
BCTQ—Function	15.00 ± 5.43	11.82 ± 4.95	0.041*	15.12 ± 8.39	12.41 ± 7.02	0.046*
Grip strength (kg)	25.54 ± 11.01	27.46 ± 12.46	*0.062*	23.43 ± 8.05	25.49 ± 10.05	*0.142*
Pinch strength (kg)	3.16 ± 1.18	3.13 ± 1.32	*0.925*	3.19 ± 2.07	2.73 ± 1.33	*0.184*
Two-point discrimination (mm)	4.00 ± 1.73	4.18 ± 1.78	*0.661*	3.71 ± 1.50	3.35 ± 1.27	*0.332*
Tinel’s sign (+/−)	11/6	6/11	0.004*	7/10	5/12	0.003*
Phalen test (+/−)	10/7	7/10	*0.646*	11/6	10/7	*0.644*

*SD* standard deviation; *BCTQ* Boston Carpal Tunnel Questionnaire; + /− positive and negative result;

*Significant difference; *p*-value > 0.05 in statistical analysis between two groups were not showed in the table. Significant values are in italics.

### Motor and sensory performance

There was no significant difference between two groups in grip strength, pinch strength and two-point discrimination *(p* > *0.05)*. After treatment, grip strength increased 1.9 kg in TENS group and 2.1 kg in ELMT group but no statistically significant difference *(p* > *0.05)* (Table 2).

### Electrodiagnostic study

There was also no significant difference with respect to the electrodiagnostic study results between two groups or within the TENS group. Significant improvements were found in the ELMT group with the mean difference ± SD of 3.55 ± 6.88 (µV) in distal sensory amplitude *(p* = *0.049)* and 6.88 ± 12.9 (m/s) in distal sensory nerve conduction velocity (NCV) *(p* = *0.043)* (Table 3).

**Table 3:** Outcomes of Electrodiagnostic study at baseline and follow up at 6 weeks.

	TENS group Mean ± SD		*p*-value within TENS group	Magnetic therapy group Mean ± SD		*p*-value within magnetic therapy group
	Baseline	Follow up		Baseline	Follow up	
Distal motor latency (mV)	6.01 ± 1.82	5.98 ± 1.99	*0.826*	5.44 ± 1.04	5.28 ± 1.13	*0.225*
Distal motor amplitude (mV)	7.90 ± 3.45	8.18 ± 3.87	*0.459*	7.91 ± 3.34	8.35 ± 2.80	*0.531*
Distal motor NCV (m/s)	51.74 ± 4.55	51.99 ± 4.57	*0.825*	53.57 ± 5.79	53.21 ± 3.16	*0.713*
Distal sensory latency (ms)	4.05 ± 0.91	4.00 ± 0.49	*0.792*	3.97 ± 0.52	3.80 ± 0.53	*0.266*
Distal sensory amplitude (µV)	9.18 ± 6.73	7.43 ± 6.26	*0.163*	7.23 ± 6.40	10.78 ± 7.78	0.049*
Distal sensory NCV (m/s)	27.65 ± 17.25	27.11 ± 15.96	*0.674*	23.21 ± 18.07	30.09 ± 15.01	0.043*
Sensory latency (P–W) (ms)	1.87 ± 1.48	2.11 ± 1.48	*0.275*	2.15 ± 1.30	2.64 ± 1.10	*0.097*
Sensory amplitude (P–W) (µV)	20.50 ± 23.96	22.34 ± 24.91	*0.646*	20.22 ± 23.59	19.61 ± 16.22	*0.897*
Sensory NCV (P–W) (m/s)	18.31 ± 14.36	19.51 ± 13.64	*0.472*	22.42 ± 13.50	24.25 ± 10.11	*0.447*
Nerve F wave mean latency (ms)	27.68 ± 3.51	28.64 ± 3.97	*0.120*	26.58 ± 2.47	27.14 ± 2.39	*0.073*
F wave persistence (%)	80.59 ± 21.06	81.77 ± 21.86	*0.798*	74.12 ± 17.70	78.82 ± 15.77	*0.308*

*SD* standard deviation; *P-W* palm to wrist

*Significant difference, *p*-value > 0.05 in statistical analysis between two groups were not showed in the table. Significant values are in italics.

## Discussion

The current study aimed to investigate the effectiveness of conventional TENS and ELMT treatments for patients with symptomatic CTS. The results showed that both TENS and ELMT treatments plus therapeutic exercises were effective to decrease symptom and improve physical function of CTS patients by 15–23%; and also decrease positive rate of Tinel’s sign. While there were no significant differences in these parameters between TENS and ELMT. The electrodiagnostic findings showed significantly improved in the sensory amplitude and NCV of the median nerve in ELMT group, whereas, no significantly changed was found in the TENS group.

Our participants in both groups performed the therapeutic exercises, including tendon gliding and nerve gliding exercises, after the electrophysical modality treatment. Previous study showed that the effectiveness of these two types of therapeutic exercises combined with conventional treatments (splint and paraffin therapy) on symptom severity and pain scale scores in patients with CTS, but no significant improvement in physical findings and nerve conduction study, or any significant differences among groups were found^25^. Most previous studies on effectiveness of various conservative treatments for patients with CTS found short-term benefits on improvement of the symptom and function^9^. BCTQ is a useful tool to assess the outcome of two categories of functional and symptom in clinical and trial settings, however, it showed only a very weak correlation to the neurophysiological and clinical severity classification system for individuals with CTS^26^. In this study, we included various aspects of the outcome measures from the validated questionnaires, clinical physical examinations and also the electrophysiological study.

In this study, we observed significant improvements of clinical symptoms and functioning scores in patients who received TENS therapy plus therapeutic exercises. The relief of acute pain and episodes is considered as a key factor for the symptom relief after conventional TENS treatment^11^. It is known to relieve pain via various mechanism, such as segmental and peripheral or neuro-mechanical mechanism^10^. Low-intensity, non-noxious TENS paranesthesia (conventional TENS) works via the segmental mechanism. Another observed effect of TENS is the peripheral blockade of afferent impulses which derived from a peripheral nerve structure^10^. Besides, other mechanisms have been proposed to explain the effect of TENS, such as anti-inflammation, surge of intracellular ATP, temporary rise in serotonin levels^27^. Similar benefits on clinical symptom from TENS treatment have been demonstrated in the previous study; however, it also showed that interferential current therapy provided a significantly greater improvement in symptom severity, functional capacity, and electrodiagnostic values than TENS therapy^28^.

The ELMT in this study not only generated an improvement in clinical symptoms and Tinel’s sign but also in electrodiagnostic results. The effectiveness was acquired due to their magnetism effects at the cellular level, as mentioned before. The result in clinical symptom improvement agreed with the study using magnetism without considering the acupuncture points in treatment of CTS^17^. Weintraub et al. used a device applied time-varying magnetic field on the volar wrist, which actually covered the PC-17, and also showed improvement in nerve conduction^18^ as our study. Also, while the device we used in this study transmitted PEMF to the selected acupoints, the inductive microamperes current (maximal average current: 10–159 μA) was generated. In study of Naeser^23^, they proved that microcurrent TENS, with subthreshold microamperes (μA) stimulation, had significant decreases in pain score, Phalen and Tinel signs, and also median nerve sensory latency after the treatment series. Unlike conventional TENS, the patient would not feel any tingling sensation from the surface electrodes. The therapeutic effects come from the increase of ATP concentrations and protein synthesis on the cellular level with the microamperes stimulation^23^. In the present study, the microamperes stimulation was delivered through the metal pointer electrode, so the patient would feel obvious tingling sensation, which would mimic the electro-acupuncture procedure. We believe it could further augment the acceptance of the treatment for the patients who seed for the assistance of the CAM therapies.

The limitation of the current study was the small sample size in this single-center trial, nevertheless, the clinical meaningful results have been demonstrated. Another limitation was only the short-term therapeutic effects were presented here. Though the improved electrodiagnostic results in ELMT group implied possible substantial neurological improvement, the long-term recovery would need further longitudinal follow-up.

In conclusion, the electroacupuncture-like magnetic therapy and conventional TENS treatment combined with therapeutic exercises were evaluated as the effective treatments to improve clinical symptoms and physical provocation sign of patents with CTS. The electroacupuncture-like magnetic therapy showed additional improvement in the electrophysiological parameters.

## Data Availability

The datasets generated during and/or analyzed during the current study are available from the corresponding author on reasonable request.
